# Brown Hare’s (*Lepus europaeus*) Histone H1 Variant H1.2 as an Indicator of Anthropogenic Stress

**DOI:** 10.1007/s00244-018-0540-z

**Published:** 2018-06-05

**Authors:** Andrzej Kowalski, Janusz Markowski

**Affiliations:** 1 Department of Biochemistry and Genetics, Institute of Biology, Jan Kochanowski University, Świętokrzyska 15, 25-406 Kielce, Poland; 2 Department of Biodiversity Studies, Didactics and Bioeducation, University of Lodz, Banacha 1/3, 90-237 Lodz, Poland

## Abstract

From the liver tissues of brown hare individuals that lived in two various habitats, i.e., the agricultural region with the predominant farms and the industrial area near a metallurgical plant, histones H1 were analyzed to compare their within and between population variability. Furthermore, because agricultural production emits mainly organic pollutants and metallurgical industry is a primarily source of inorganic contaminations, we wanted to check how the brown hare individuals are sensitive for both agents. Among brown hare H1 histones, the histone H1.2 was determined as heterogeneous due to its varied mobility in two-dimensional SDS–polyacrylamide gel. The obtained electrophoretic patterns contained differently moving single spots of histone H1.2 and also its double spots have a similar rate of electrophoretic mobility. Based on this, two homozygous phenotypes (slowly migrating 2a and faster moving 2b) and a heterozygous phenotype (2a2b) was distinguished. The relatively low variable (CV < 0.25) and comparably abundant (*p* > 0.05) histone H1.2 homozygous phenotypes form a heterozygous phenotype in a similar proportion, at a ratio approximating 0.5. Although the brown hare population originating from agricultural area displayed a slight excess of heterozygous individuals 2a2b (*F* = − 0.04), it was conformed to the Hardy–Weinberg assumption (*χ*^2^ = 0.035, *p* = 0.853). Compared with this population, a sevenfold reduced frequency of the phenotype 2b and above tenfold increase of a heterozygosity (*F* = − 0.53) was observed in the brown hare population inhabiting the vicinity of metallurgical plant. Therefore, this population did not fit to the Hardy–Weinberg law (*χ*^2^ = 5.65, *p* = 0.017). Despite the negligible genetic differentiation (*F*_ST_ = 0.026) between brown hare populations inhabiting areas with different anthropogenic pressure, a statistically significant difference in the distribution of their phenotypes (*χ*^2^ = 6.01, *p* = 0.049) and alleles (*χ*^2^ = 6.50, *p* = 0.013) was noted. The collected data confirm that the brown hare species is sensitive for environmental quality and may serve as a good indicator of habitat conditions related to both organic pollution emitted by agricultural activities (PIC = 0.48) and inorganic contamination originating from metallurgical processes (PIC = 0.49). These difference in the environmental quality might be assessed by estimation of genetic variability among the brown hare populations, based on the phenotypes distribution of histone H1 variant H1.2, the protein that was not so far employed as a molecular marker of anthropogenic stress.

The European brown hare*, Lepus europaeus*, originating from the steppes of Eurasia was spread out in the crop regions of Europe (Averianov et al. 2003) becoming an important mammalian small game species (Pielowski 1976; Chapman and Flux 1990). The regular monitoring of hunting bags allowed scientists to follow the long-term trends in abundance and natural fluctuations of local brown hare populations. This enabled a decline of the brown hare in the early 1960s and also a systematic decrease of its number in recent decades throughout Europe (Tapper and Parsons 1984; Mary and Trouvilliez 1995; Pielowski 1976). As a result, the brown hare species has been included in the International Union for Conservation of Nature (IUNC) Red List and considered to be a species of low-risk extinction under Appendix III of the Berne Convention on the Conservation of European Wildlife and Natural Habitats (Mitchell-Jones et al. 1999).

A significant reduction of the whole brown hare population caused tremendous research interest, and therefore, various reasons explaining this phenomenon have been suggested. A probable cause of the individual decrease in the brown hare population pointed to the agricultural intensification and reduction of habitat heterogeneity, the fields enlargement, and decreased crop diversity, as well as the landscape fragmentation by roads and climate changes (Smith et al. 2005). Likewise, an increase in the predator numbers and infectious diseases, including the European Brown Hare Syndrome (EBHS) occurring in Poland (Kałuzinski and Pielowski 1976; Panek and Kamieniarz 1999; Panek et al. 2006) and other European countries (Edwards et al. 2000; Schmidt et al. 2004), were considered as the factors that favor reduction of the brown hare abundance. Since the 1980s, the European brown hare has become a target of genetic studies. Research focused mainly on testing whether such a decline of brown hares might be associated with diminished overall fitness linked to the reduction of genetic variability. As the first molecular markers, the allozymes have been used extensively to describe the genetic structure of brown hare populations (Hartl et al. 1990, 1992, 1994; Suchentrunk et al. 2000; Ben Slimen et al. 2008). These studies revealed that brown hare populations are locally subjected to genetic drift and also on a low exchange of genes between the populations (Wincentz 2009). In addition, the brown hare allozymes can have different metabolic functions (Mitton and Lewis 1989); therefore, a selection can act on the allozyme frequencies (Mitton and Koehn 1975; Powers and Place 1978; Powers et al. 1986; Mitton 1993). As shown by Markowski et al. (1990), the haptoglobin can be used as an informative marker of the brown hare health status. An influence of contamination on brown hare population genetic structure was documented previously as linked to the physiological processes (Pav and Zahradnikova 1987; Paukert 1988). Thus, investigations of the factors that may affect brown hare genetic variability evoked by the man-induced environmental changes are still needed. The current study was undertaken to determine if brown hare populations are diverse in the agricultural and industrial regions contaminated by organic and inorganic pollutants, respectively. A genetic variation between the brown hare populations, which reflects a susceptibility of animals to the environmental changes, was assessed based on the polymorphic variability of histone H1.2, a representative of histone H1 mammalian somatic subtypes.

To monitor the shifts of allele frequency at the brown hare polymorphic loci, the varied methods using a set of molecular markers are usually adopted. Besides the above-mentioned allozymes, the mitochondrial and microsatellite DNA is widely used for estimation of brown hare population status (Soós and Kusza 2015). However, suitable for this purpose also may be the other indicators, such as H1 histone proteins, which seems to be effective for the evaluation of brown hare population structure and its variations caused by anthropogenic activity. The histone H1.2 presented in this work seems to be informative in monitoring the regions contaminated by chemical agents of different origins. Among them are gas and heavy metals pollution emitted especially from works of heavy industries and contamination by macro elements, which are predominantly accumulate in the plants and soil as a consequence of agricultural production (Malinowska 2004).

Histone H1, a ubiquitous protein that determines chromatin structure and modulates its activity (Parseghian 2015; Kowalski and Pałyga 2016), is highly heterogeneous (Kowalski and Pałyga 2012a). By interaction with DNA (Izzo and Schneider 2015) and partnering proteins (McBryant et al. 2010), the individual histone H1 variants regulate various cell activities, including gene expression and cell cycle progression as well as biogenesis and metabolism of the RNA (Kalashnikova et al. 2016). The nonallelic histone H1 variants, which on average comprise ten forms in mammals and birds (Kowalski and Pałyga 2017), are polymorphic. In birds, histone H1 allelic variants are widespread. Among the nine histone H1 nonallelic forms, only one variant (H1.c’) has not been recognized to date as polymorphic (Kowalski and Pałyga 2012a). Whereas the functions of avian histone H1 allelic variants are not clearly determined, their potential link with the specific phenotypic effects (Kowalski and Pałyga 2014) and some physiological traits of the organisms (Kowalski et al. 2015) has been reported. The rarely detected histone H1 polymorphic subtypes in mammals include the rabbit histone H1.4 (Pałyga 1990) and mouse histone H1.S (Zweidler 1984) as well as the human histone H1.2 and H1.4 (Sarg et al. 2005). While a functional importance of mammalian histone H1 allelic variants remains unknown, the last findings of Flanagan et al. (2016) revealed that the mouse histone H1 polymorphic variants, H1.1 and H1.5, differ in their interaction with chromatin. Thus, histone H1 allelic variants may have a specialized role related to their individual impact on the modulation of chromatin structure and function. Likewise, the brown hare histone H1.2 presented in this work is rare histone H1 polymorphic variant whose phenotypic heterogeneity may be useful for evaluation of the changes that take place in populations living in the regions exposed to different environmental pollutants.

## Materials and Methods

### Animal Material

**Fig. 1:**
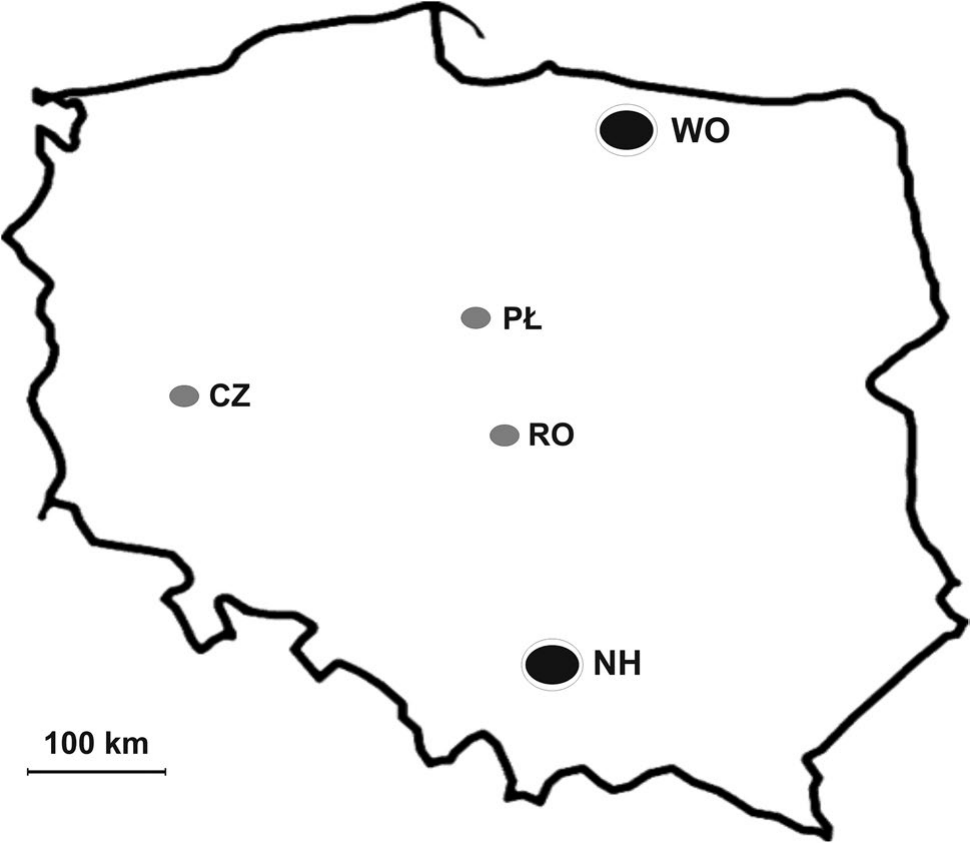
A map of Poland presenting the hunting areas, i.e., Wopławki (WO) in the Mazurian Lakes and Nowa Huta (NH) in the Małopolska province (black points), from which the brown hares were analyzed in this work and the regions from which the brown hares were caught in the same hunting seasons, i.e., Czempiń (CZ) in the Leszno Lakes as well as Płock (PŁ) in the Mazovian industrial region and Rogów (RO) in the Mazovian agricultural space (grey points)

The liver tissue samples of brown hares were collected during the hunting seasons, years 1993–1995, from two areas representing different physiographic regions in Poland, i.e., from area of Wopławki in the Mazurian Lakeland with predomination of agricultural farms and from industrial region of Małopolska province in the immediate vicinity of metallurgical plant in the Nowa Huta (Fig. 1). The culled hares were immediately weighed, to the nearest 0.1 kg, and dissected for inspection of sex organs to determine their gender. The livers and eye balls were extracted and put into the zip lock plastic bags in which they were transported to the laboratory in ice. The eye balls were left for 3 days in a 10% formalin. Then, the lenses were excised and cleaned thoroughly with deionized water. After dried in an oven at 80 °C for one night and cooled in a desiccator, the lenses were weighed to the nearest of 0.1 mg on a microbalance in pairs. Determination of the animal age based on the lens weight is possible, because the growth of lens lasts to grow up to the death (Lord 1959; Augusteyn 2008) and, hence, the dry mass of the eye lens has commonly been used to estimate the age of mammalian species (Lord 1959; Friend 1967; Dapson 1980; Augusteyn 2014). The individuals with a dry weight of the lenses less than 275 mg were classified as juveniles (Andersen and Jensen 1972; Cabon-Raczynska and Raczynski 1972; Suchentrunk et al. 1991). The liver samples were perfused with a cold solute on containing 0.13 M NaCl, 0.5 mM KCl, and 0.8 mM MgCl_2_ supplemented with 0.1 mM phenylmethylsulfonyl fluoride (PMSF) and stored at − 20 °C until H1 histones were isolated.

### Isolation of Nuclei and Extraction of Histone H1

Liver nuclei were prepared by a modified method of Bush and Daskal (1977), with the use of 0.1% Triton X-100 for lysing the cells and a subsequent sedimentation of nuclei through 1.75 M sucrose buffered with a solution containing 0.25 mM KCl, 0.5 mM MgCl_2_, 0.5 mM Tris–HCl, pH 7.5.

Histones H1 were extracted according to the procedure of Neelin et al. (1995), first with 1 M perchloric acid solution and then with the perchloric acid in the concentration of 0.5 M. The combined supernatants were precipitated with 20% trichloroacetic acid and washed twice with the acetone acidified with HCl (250:1 v/v) and doubly with acetone alone. Dried histone H1 preparations (1 mg) were added to a solution (100 mL) containing 8 M urea, 0.9 M acetic acid, and 10% 2-mercaptoethanol to prepare the histone H1 samples for electrophoresis.

### Electrophoresis of Histone H1

Electrophoretic separation of H1 histones was done as described by Kowalski and Pałyga (2012b). Both first dimension (acetic acid-urea) and second dimension (SDS) electrophoretic gels were 24-cm long. First dimension electrophoretic gels were prepared with 15% acrylamide, 0.5% methylenebisacrylamide, and 8 M urea. The second dimension electrophoretic gels were composed of 13.5% polyacrylamide and 0.1% SDS. Proteins were stained with the Coomassie Blue R-250, in the concentrations of 0.05 and 0.0035%, mixed with the mixture of acetic acid-propanol-2 (10–25%) and destained with 10% acetic acid solution. After resolution of proteins in the first dimension, the gel fragments containing the stained H1 histone protein bands were cut out and equilibrated (2 × 15 min) for the second dimension in the buffer containing 100 mM Tris-base pH 6.8, 10% glycerol, 2.1% SDS, and 2% 2-mercaptoethanol.

### Gel Images Processing and Measurements of Histone H1.2 Phenotypes Quantity

The obtained electrophoretic patterns of H1 histones were recorded with the gel imaging system Doc-Print II (Vilber Lourmat) and processed by the software ImageJ 1.44c (www.rsbweb.nih.gov/ij). A raw integrated density, which indicate a sum of the values of the pixels in the selected gel area, was measured to evaluate an abundance of histone H1.2 phenotypes protein spots. The measurements were repeated for the seven preparations (*n* = 7) of each histone H1.2 phenotype.

### Statistical Evaluation

A significance of difference between the levels of histone H1.2 phenotypes was evaluated with the Student’s *t* test. The coefficient of variation for histone H1.2 phenotypes was calculated as a ratio of standard deviation and the mean, assuming that the value less than 0.25 corresponds to the low variability. Testing for fit of brown hare populations to the Hardy–Weinberg equilibrium was done with the use of Chi square (*χ*^2^) test of goodness-of-fit. A difference among the brown hare age and sex as well as between the levels of histone H1.2 alleles and phenotypes was evaluated with the use of Chi square (*χ*^2^) test of homogeneity. In all tests, a *p* value <0.05 was regarded as statistically significant for rejecting the null hypothesis. The genetic differentiation of brown hare population was evaluated by Wright’s *F* statistics indices (Excoffier 2007). The polymorphic information content (PIC) of the histone H1.2 locus was evaluated through the allelic frequency with the use of formula adopted by Anderson et al. (1993).

## Results

### Characteristics of Brown Hare Histone H1.2 Heterogeneity

**Fig. 2:**
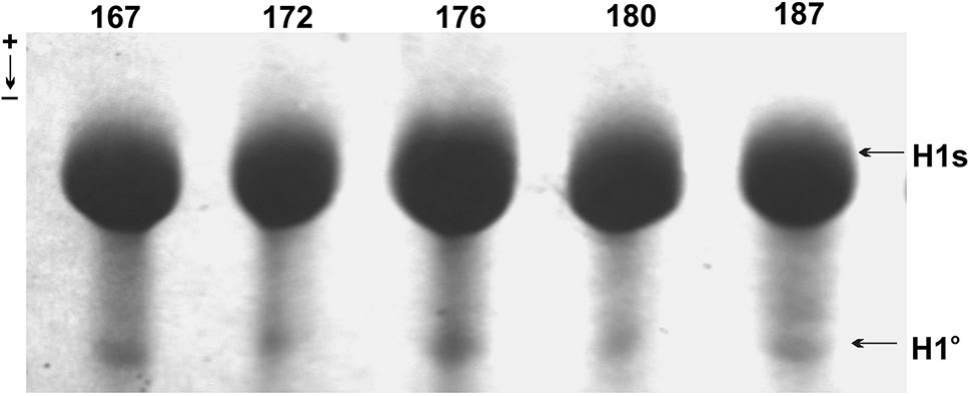
The electrophoretic pattern of brown hare histones H1 resolved in the first dimension, acetic acid-urea polyacrylamide gel. The slowly migrating intense bands of H1 subtypes (H1s) and the faster moving minute bands of subtype H1 (H1^o^) that belong to different individuals, numbered 162, 172, 176, 180, and 187, are depicted

**Fig. 3:**
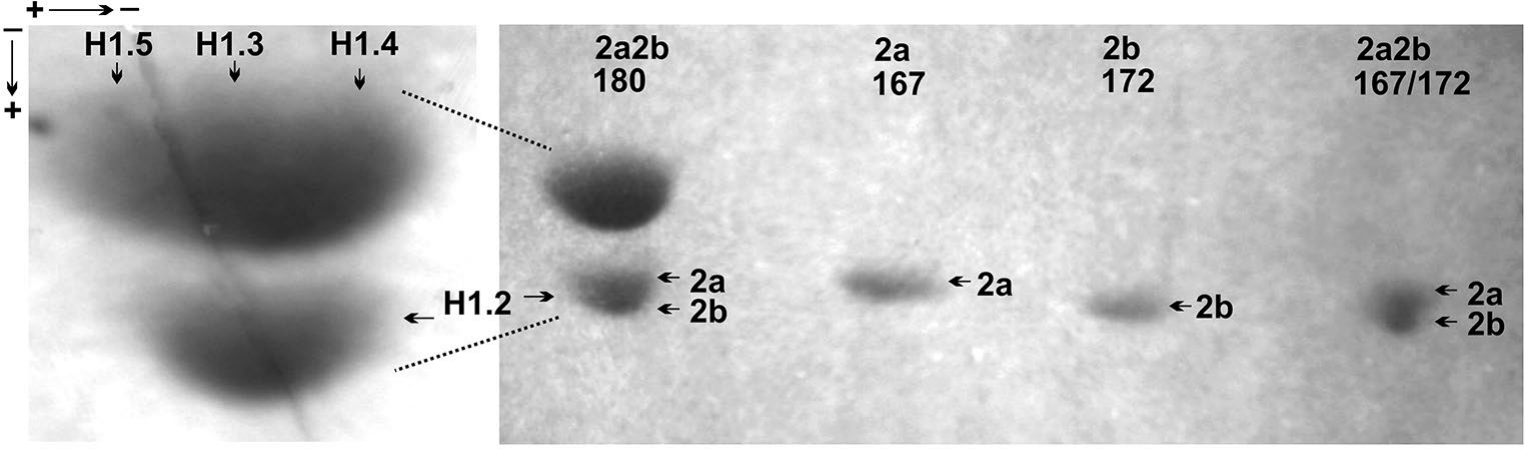
The second dimension, SDS polyacrylamide gel, electrophoretic pattern of brown hare histone H1. On the left, three slow migrating protein spots of subtype H1.3, H1.4, and H1.5 and the fast migrating protein spot of subtype H1.2. On the right, the variability of histone H1.2 reflected by the presence of separately migrating phenotype 2a2b (individual 180), 2a (individual 167), and 2b (individual 172) and a joint migration of the co-electrophoresed phenotype 2a (individual 167) and 2b (individual 172)

Histone H1 preparations obtained from brown hare livers in the perchloric acid soluble fraction were initially analyzed in the first dimension acetic acid urea polyacrylamide gel. As seen in the Fig. 2, this electrophoretic technique allows to separate the H1 histones into two fractions only, i.e., a slow migrating bulk of unresolved histone H1 subtypes protein bands and a faster moving individual band of the histone H1.0. However, the more precisely separated H1 histones were visible in the second dimension SDS polyacrylamide gel (Fig. 3), in which besides the low mobile histone H1 variants, H1.3, H1.4, and H1.5, a far migrating histone H1.2 also was present. According to the differential rate of histone H1.2 electrophoretic migration, the homozygous phenotypes (2a and 2b) formed by single protein spots and a heterozygous phenotype (2a2b) composed of double spot was identified. The co-electrophoresed protein spots belonging to the homozygous phenotypes (2a and 2b) were found as possessing similar in-gel location to the protein spots constituting a heterozygous phenotype 2a2b. Such an electrophoretic pattern confirmed the presence of histone H1.2 heterozygotes (Fig. 3). Quantitative evaluation of the levels of histone H1.2 homozygous phenotypes protein spots revealed their similar amount (*p* = 0.571) and a comparable share in the heterozygous phenotype (Table 1). Also, a similar proteins abundance was characteristic for both heterozygotes and their co-electrophoresed counterparts (*p* = 0.415). Based on the calculated values of the coefficient of variation, not exceeding 0.25, a relative variability of histone H1.2 phenotypes is low (Table 1). Thus, brown hare histone H1.2 is a polymorphic protein determined by the presence of three phenotypes (2a, 2b, and 2a2b), which are coded by two codominant alleles (*2a* and *2b*) at a locus.

**Table 1:** Abundance of histone H1.2 phenotypes detected in the two-dimensional electrophoretic gel patterns

Histone H1.2 phenotype	2a^a^	2b^b^	2a2b^x^	co-2a2b^y^
Mean ± standard deviation	1062.3 ± 112.5	1088.4 ± 88.1	1972.6 ± 211.7	1969.4 ± 171,7
Coefficient of variation	0.105	0.081	0.107	0.087

The level of histone H1.2 phenotypes protein spots resolved separately (2a and 2b) and co-electrophoresed in common (co-2a2b) were measured as an integrated density, which is a sum of the values of the pixels in the selected gal area, with the ImageJ processing program for separate individuals (*n* = 10) belonging to a given phenotype

^a,b^*p* = 0.571

^x,y^*p* = 0.415

### Histone H1.2 Genotypes Variability Within and Between Brown Hare Population

Determination of the brown hare age structure indicates that the adult individuals constitute a major part, i.e., 73.6% of the population while a share of sexes is almost the same and amount to 48.6 and 51.4% of males and females, respectively. Thus, no statistically significant difference between juvenile and adult individuals (*Χ*^2^= 3.54, *p* = 0.17) as well as between both groups of sexes (*Χ*^2^= 2.92, *p* = 0.23) has been detected in the brown hares originating from all regions in which they were caught (Table 2). The analyses of brown hare populations structure originating from the regions exposed to different environmental pollutants is presented in Table 3. While the brown hare population originating from agricultural area of Wopławki conformed to the Hardy–Weinberg proportions (*Χ*^2^= 0.035, *p* = 0.853), a population living in the vicinity of metallurgical plant in the Nowa Huta significantly differ from the Hardy–Weinberg expectation (*Χ*^2^= 5.65, *p* = 0.017). A severe shortage of the homozygous 2b individuals (frequency 0.05) was detected in the amount of seven times less compared with the same individuals from the area of Wopławki (frequency 0.35). Besides, the surplus of heterozygous 2a2b individuals, exemplified by a negative value of within population inbreeding coefficient (*F*_s_ = − 0.53; Table 4), also was characteristic for this population. Thus, a statistically significant difference in the distribution of histone H1.2 alleles (*Χ*^2^ = 6.50, *p* = 0.013) and phenotypes (*Χ*^2^= 6.01, *p* = 0.049) frequency was found between populations. To determine differentiation of brown hare populations, the Wright’s *F*-statistics was used as a measure of their genetic variation. The different values of individuals (*H*_I_ = 0.625) and subpopulations (*H*_S_ = 0.485) heterozygosity confirmed deviation from the Hardy–Weinberg assumption, which also was identified in relation to the negative value of *F*_IS_ (− 0.288) and *F*_IT_ (− 0.255) indices calculated from observed and expected heterozygosity, respectively (Table 4). However, the value of *F*_ST_ amounting 0.026 (Table 4) indicate a negligible genetic differentiation between populations and shows that only approximately 3% of the total genetic variation concerns a population difference and 97% correspond to the difference between individuals. The relatively low values of F_ST_ among the populations may be caused by a lack of equilibrium between migration and drift. It also seems that brown hare histone H1.2 can be used as an reasonably informative marker of environmental changes evoked by both agricultural pollution (PIC = 0.48) and petrochemical contamination (PIC = 0.49; Table 4). The most suitable marker of pollution-induced changes is probably more sensitive variant H1.2b (phenotype 2b), frequency of which was strongly reduced, i.e., seven times lower frequency, in the population inhabiting the industrialized region.

**Table 2:** Age and sexes structure of whole collected brown hare individuals from agricultural, i.e. Czempiń, Rogów and Wopławki, and industrial, i.e. Płock and Nowa Huta, areas

Histone H1.2 phenotype	Number of individuals (observed/expected)			
	Age		Sexes	
	Juvenile^a^	Adult^a^	Male^b^	Female^b^
2a	5/4.09	10/10.9	11/9.24	8/9.76
2b	1/3.81	13/10.18	4/6.82	10/7.18
2a2b	12/10.09	25/26.9	21/19.95	20/21.05

^a^*Χ*^2^ = 3.54, *p* = 0.17

^b^*Χ*^2^ = 2.92, *p* = 0.23

**Table 3:** Histone H1.2 phenotypes and alleles frequency of brown hare population living in the agricultural region of Wopławki and around the metallurgic industry in Nowa Huta

Phenotype^a^	2a	2b	2a2b	Allele^b^	*2a*	*2b*
*Wopławki*						
Number of individuals (observed/expected)	3/3.2	7/7.2	10/9.6	Frequency of allele	0.4	0.6
Frequency of phenotype (observed/expected)	0.15/0.16	0.35/0.36	0.5/0.48			
HWE *Χ*^2^ = 0.035, *p* = 0.853						
*Nowa Huta*						
Number of individuals (observed/expected)	4/6.6	1/3.6	15/9.8	Frequency of allele	0.575	0.425
Frequency of phenotype (observed/expected)	0.2/0.33	0.05/0.18	0.75/0.48			
HWE *Χ*^2^ = 5.65, *p* = 0.017						

^a^The phenotypic (*Χ*^2^ = 6.01, *p* = 0.049)

^b^Allelic (*Χ*^2^ = 6.50, *p* = 0.013) diversity between populations

**Table 4:** Genetic variation among the brown hare populations

Population	Heterozygosity		Local inbreeding coefficient (*F*_s_)	Wright’s *F*-statistics indices			Polymorphic information content (PIC)
	Observed	Expected		*F*_IS_	*F*_ST_	*F*_IT_	
Wopławki	0.5	0.48	0.04	− 0.028	0.026	− 0.255	0.48
Nowa Huta	0.75	0.49	− 0.53				0.49

## Discussion

As already reported, the histone H1 has multiple subtypes in various organisms (for a review, see Parseghian 2015; Kowalski and Pałyga 2016; Fyodorov et al. 2017). In the set of mammalian H1 histones, there are regular variants present in the somatic cells (H1.1, H1.2, H1.3, H1.4, H1.5, H1.0, and H1.10) and the variants characteristic for specialized cells, such as sperm (H1t) and oocyte (H1oo) (Happel and Doenecke 2009). Histone H1 polymorphic variants fluctuate in a population. In the rabbit breeds, a rare histone H1.4 (H1e) phenotype B was found as occurring at a frequency that ranges from 0.10 to 0.28 only (Pałyga 1990). However, the changes in the distribution of histone H1 polymorphic variants are well known mainly in the avian H1 histones. For example, the duck histone H1 variant H1.a2 was shown as missing in the conservative flocks, in contrast to the variant H1.a1 commonly present in all strains tested (Górnicka-Michalska et al. 2014). Likewise, the phenotype b2 and z2 of histone H1.b and H1.z, respectively, was not detected in general in the production and conservative groups of the duck (Kowalski and Pałyga 2014). A disproportion of allele frequency in the histone H1.b, H1.d, and H1.z was revealed between quail control line and the line selected for a high yolk cholesterol content (Kowalski et al. 2015). Furthermore, a complete lack of histone H1 phenotype b1b2 and c1c2 in the guinea fowl strains (Kowalski et al. 2011) and an absence of histone H5 phenotype ab in the pheasant population (Kowalski 2016) was observed after a relocation of bird individuals from natural habitat to the breeding. Thus, the fluctuation of histone H1 polymorphic variants is related to the differences in animal living places, i.e., natural versus breeding habitats, and also linked to some physiological traits of the organisms. It seems, however, that a scope of the factors that influence on the distribution of histone H1 phenotypes and alleles might be broader and corresponding to the environmental heterogeneity. An example is the brown hare histone H1.2, whose polymorphic variants were identified in the current study as differently arranged in the area polluted by agricultural and metallurgic activity. The evidence for a selective value of H1 histone molecular polymorphism was shown in a natural population of the wild leguminous plant *Vicia unijuga* inhabiting the territory and surroundings of Novosibirsk. The distribution of four alleles belonging to one of the histone H1 subtype revealed a radial cline of their pattern, which was likely due to some of man-caused factor, presumably insecticide, that has been used for a period of time not exceeding the 25 years (Berdnikov et al. 1992).

The toxicants that are produced by agricultural farms and industrial plants are different (Malinowska 2004). The nitrogen derivatives, i.e., nitrous oxide, nitrogen oxides, and ammonia, are primarily released from fertilizers in the agricultural production (Parris 2011; Savci 2012) and the various mixtures of heavy metals, i.e., cadmium, nickel, zinc, and lead, are the prevalent side products of metallurgic processes (Dai et al. 2015; Mizerna 2106). Although there are no data about contamination released by agricultural activity in the region of Wopławki, a higher concentration of nitrates has been observed in the water of lakes located in the same region (Zieliński et al. 2013; Mioduszewski 2015). Likewise, a determination of pollutions in the close vicinity of Wopławki indicated a higher content of magnesium, nitrogen, and phosphorus compounds in the places lying near the agricultural areas (Domska et al. 2010). However, as shown by Sobolewski (2016), a predominant number of Masurian lakes possess transparent water with low level of the nitrogen compounds. A much higher content of the heavy metals, i.e., zinc, cadmium, and lead, was detected in the soil samples around steel plant in the Nowa Huta (Lenart and Wolny-Koladka 2013). Similarly, the same agents were highly concentrated in the tissues of animals, i.e., goat and sheep (Mundała et al. 2016), bank vole (*Myodes glareolus*) (Damek-Poprawa 2002; Topolska et al. 2004), and yellow-necked mice (*Apodemus flavicollis*) (Damek-Poprawa 2002), which originated from this area. Thus, the brown hare individuals from populations living in the contaminated areas might be variously sensitive for such agents, serving simultaneously as an indicators of their incidence. These environmental changes might be monitored with the help of histone H1 variant H1.2, which could be considered as a reasonably informative marker (Botstein et al. 1980) of anthropogenic activity related to the pollutions. It seems that especially vulnerable for potential toxicants released by metallurgic production is a phenotype 2b, very poorly represented in population living in the vicinity of steel plant, in contrast to the phenotype 2a that is similarly abundant in both agricultural and industrial space. Such a change in the histone H1.2 phenotypes arrangement implies that histone H1 polymorphic variants might not be functionally equivalent and, hence, could act individually to regulate the chromatin-dependent physiological processes.

The environmental stress associated with habitat pollution can affect population structure by increasing and/or decreasing its genetic variability. As a consequence, a shift in the distribution of tolerant phenotypes and correlation between the contaminants and allele frequency may occur. The susceptibility to pollutants reduces a viability of organisms and weakens the survival of a species (Van Straalen and Timmermans 2002). The results that concerns mammals are, however, contradictory. While Berckmoes et al. (2005) and Mikowska et al. (2014) showed no correlation between genetic variability and a level of metal pollution in the wood mouse (*Apodemus sylvaticus*) and bank vole (*M. glareolus*), respectively, the data presented by Mussali-Galante et al. (2013) indicate a significant negative correlation between genetic diversity and metal concentration in the plateau mouse (*Peromyscus melanophrys*). The brown hare species is sensitive to many factors that influence a density of its population. The landscape change corresponding to expanded urbanization and agriculture intensification (Edwards et al. 2000; Lundström-Gillieron and Schlaepfer 2003), as well as the activity of hunters and predators (Smith et al. 2005), especially influences on the brown hare demographic variation and cause a rearrangement of the population (Hartl et al. 1990; Ben Slimen et al. 2008; Zhelev and Ninov 2014). An analysis conducted in the current study demonstrates that the brown hare population is variously sensitive for anthropogenic factors of different origin and show that the brown hare individuals might be deemed as a proper indicator of environmental contamination.

In summary, the results presented in this work show that the polymorphic histone H1.2 is informative in the monitoring of genetic variations in the brown hare population, which undergo a rearrangement in relation to anthropogenic changes correlated with different environmental pollutants.
